# Reactive Oxygen Species Are Required for Maintenance and Differentiation of Primary Lung Fibroblasts in Idiopathic Pulmonary Fibrosis

**DOI:** 10.1371/journal.pone.0014003

**Published:** 2010-11-16

**Authors:** Marialuisa Bocchino, Savina Agnese, Evelina Fagone, Silvia Svegliati, Domenico Grieco, Carlo Vancheri, Armando Gabrielli, Alessandro Sanduzzi, Enrico V. Avvedimento

**Affiliations:** 1 Dipartimento di Medicina Clinica e Sperimentale, Sezione di Malattie dell'Apparato Respiratorio, Università Federico II, Napoli, Italy; 2 Dipartimento di Biologia e Patologia Cellulare e Molecolare “L. Califano”, Università Federico II, Napoli, Italy; 3 Dipartimento di Medicina Interna e Specialistica, Sezione di Malattie Respiratorie, Università di Catania, Catania, Italy; 4 Dipartimento di Scienze Mediche e Chirurgiche, Sezione di Clinica Medica, Università Politecnica delle Marche, Ancona, Italy; Comprehensive Pneumology Center, Germany

## Abstract

**Background:**

Idiopathic pulmonary fibrosis (IPF) is a progressive and fatal illness whose pathogenesis remains poorly understood. Recent evidence suggests oxidative stress as a key player in the establishment/progression of lung fibrosis in animal models and possibly in human IPF. The aim of the present study was to characterize the cellular phenotype of fibroblasts derived from IPF patients and identify underlying molecular mechanisms.

**Methodology/Principal Findings:**

We first analyzed the baseline differentiation features and growth ability of primary lung fibroblasts derived from 7 histology proven IPF patients and 4 control subjects at different culture passages. Then, we focused on the redox state and related molecular pathways of IPF fibroblasts and investigated the impact of oxidative stress in the establishment of the IPF phenotype. IPF fibroblasts were differentiated into alpha-smooth muscle actin (SMA)-positive myofibroblasts, displayed a pro-fibrotic phenotype as expressing type-I collagen, and proliferated lower than controls cells. The IPF phenotype was inducible upon oxidative stress in control cells and was sensitive to ROS scavenging. IPF fibroblasts also contained large excess of reactive oxygen species (ROS) due to the activation of an NADPH oxidase-like system, displayed higher levels of tyrosine phosphorylated proteins and were more resistant to oxidative-stress induced cell death. Interestingly, the IPF traits disappeared with time in culture, indicating a transient effect of the initial trigger.

**Conclusions/Significance:**

Robust expression of α-SMA and type-I collagen, high and uniformly-distributed ROS levels, resistance to oxidative-stress induced cell death and constitutive activation of tyrosine kinase(s) signalling are distinctive features of the IPF phenotype. We suggest that this phenotype can be used as a model to identify the initial trigger of IPF.

## Introduction

Idiopathic pulmonary fibrosis (IPF) is a progressive and lethal lung disorder with a mean survival of 3–6 years from the onset of symptoms. Histology of IPF shows the features of usual interstitial pneumonia with patchy distribution of fibrosis adjacent to fibroblastic foci (FF) [1]. IPF appears to be an “epithelial-fibroblastic disease” resulting from recurrent epithelial injury and abnormal wound repair [2]. FF are composed of migrating and proliferating fibroblasts and of differentiated myofibroblasts accounting for extra-cellular matrix deposition slowly altering the alveolus structure. This explains the progressive and irreversible IPF nature and the prognostic value of the fibrosis extent [3], [5].

IPF pathogenesis is unknown and the role of inflammation remains controversial, since anti-inflammatory treatment does not produce significant benefit against the disease progression. Inflammation is likely the triggering event for the initiation of fibrosis; eventually, fibrosis self-maintains and progresses by an unknown process [6], [7]. Recent studies have emphasized the role of oxidative stress as the molecular basis of lung fibrosis. Reactive oxygen species (ROS) are key players in the establishment/progression of pulmonary fibrosis in animal models and possibly in human IPF [8]–[11]. There is evidence of disruption of the normal oxidant/antioxidant balance in the lungs of IPF patients. Deficiency of antioxidants, including glutathione and superoxide dismutase, has been found in the lower respiratory tract of IPF patients, while high levels of myeloperoxidase are associated with epithelial injury in the fibrotic lesions [12]–[14].

Fibroblasts and myofibroblasts are recognized as the effector cells in normal wound healing and in the development of tissue fibrosis [15]. Although the interaction of these cells with a large spectrum of growth factors involved in tissue remodelling has been extensively investigated in IPF, their relationship with oxidative stress remains poorly clarified. The aim of the present study was to characterize the baseline cellular phenotype of fibroblasts derived from IPF patients and to identify molecular targets underlying this phenotype.

## Materials and Methods

### Ethics Statement

The study was approved by the Institutional Review Board for biomedical activities of the Universities of Naples, Ancona and Catania and by the Ethics Committee of the Monaldi hospital, Naples, all in Italy. Patients provided written informed consent.

### Cell culture

Primary lines of human lung fibroblasts were established by using an outgrowth from explant following the method described by Jordana *et al.* [16]. IPF cell lines were obtained from 7 patients affected by IPF (age range 48–60 y), undergoing surgical lung biopsy for diagnosis. Control fibroblasts were derived from normal lung tissue of 4 patients with tumour-free areas of lung lobes with early stage bronchial carcinoma (age range 45–55 y). Cells were grown under standard conditions at 37°C in 5% CO_2_ in DMEM with 1 g/l glucose supplemented with 10% fetal bovine serum (FBS), 2 mM L-glutamine, 100 IU/ml penicillin and 100 µg/ml streptomycin, and used at 80–90% confluence at different culture passages. To distinguish experiments with different culture timing, cells used within passage VI were arbitrary referred as “early passage” while cells used later on through passages IX-XI were referred as “late passage”. Overall, early passage cells were defined as cells within 20–25 population doublings (PD), while late passage cells had a PD greater than 45. All reagents were purchased from GIBCO (Scotland, UK). All experiments were performed in duplicate.

### Cell treatments

Reagents used for cell treatments included: 1. H_2_O_2_ (Sigma, St. Louis, MO, USA); 2. the recombinant human transforming growth factor (rhTGF)-β1 (Calbiochem, Merck Darmstadt, Germany); 3. the TGF-β receptor ALK5 inhibitor SB-431542 (Sigma); 4. the ROS scavenger, N-acetylcisteine (NAC, Sigma); and 5. the general oxidase inhibitor diphenyleneiodonium (DPI, Calbiochem). Additional treatments included the MEK kinase inhibitor UO126 and the receptor tyrosine kinase (RTKs) inhibitors AG1296 (PDGF-R inhibitor) and AG1478 (EGF-R inhibitor) (Calbiochem) [17]. Dose-response curves were performed with all reagents.

### Cell proliferation assay

Proliferation assays were performed in 96-well microtiter plates. Cells were cultured at a density of 1×10^3^ viable cells per well for 48 h and pulsed for additional 24 h with 0.25 µCi/well of [^3^H] thymidine (Amersham, Pharmacia Biotech Buckinghamshire, UK). Cells were harvested on glass-fiber filters using a Tomtec (Orange) 96-well cell harvester and counted in a 1205 Betaplate liquid scintillation counter (Wallac). Results obtained from three wells were expressed as mean cpm±SD.

### Cell death assay

Non adherent and trypsin-treated adherent cells were collected and washed twice with cold PBS. Cell pellets were re-suspended in 1 ml of propidium iodide (PI) staining solution (PI 5 µg/ml). Samples were kept at room temperature (RT) in the dark and immediately analysed by flow cytometry (Cyan DAKO Cytomation, Colorado, USA). For each sample 30.000 events were acquired and PI positive cells were detected at a wavelength of 635 nm. Data analysis was carried out with the Summit software (version 4.3, DAKO).

### Senescence β-galactosidase assay

Medium was removed from sub-confluent cultures. Cells were fixed with 3% formaldehyde for 10 min at RT, washed and incubated overnight at 37°C in staining solution containing 1 mg/ml X-Gal (5-bromo-4-chloro-3-indolyl-β-D-galactopyranoside), purchased from Promega (Madison, WI, USA), 40 mM citric acid/sodium phosphate (pH 6.0), 5 mM potassium ferrocyanide, 5 mM potassium ferricyanide, 150 mM NaCl, and 2 mM MgCl_2_. Cells were checked under a microscope for development of blue colour and the mean percentage of β-galactosidase-positive cells was calculated by counting 3 representative fields.

### RNA purification and real-time PCR

Total RNA was extracted with TRIzol reagent according to the manufacturer's protocol (Sigma) and two micrograms were reverse-transcribed with the Omniscript RT kit (Qiagen, Milan, Italy) using random primers (1 µM) at 37°C for 1 h. Real time PCR was performed in triplicate in 20 µl reaction volumes using the Power SYBER Green PCR Master Mix (Applied Biosystems, Foster City, CA, USA). PCR primers (0.2 µM) for human A2 type-I collagen used were: sense 5′-TCTGGAGAGGCTGGTACTGC-3′ and anti-sense 5′-GAGCACCAAGAAGACCCTGA-3′. PCR primers (2 µM) for human transforming growth factor (TGF)-β used were: sense 5′-ACTACTACGCCAAGGAGGTCAC-3′, anti sense 5′- TGCTTGAACTTGTCATAGATTTCG-3′. 18S RNA was used as reference (sense 5′-TCC CCA TGA ACG AGG AAT TC-3′ and anti-sense 5′-GTG TAC AAA GGG CAG GGA CTT-3′). All primers were purchased from Invitrogen (CA, USA). Real time PCR reactions were carried out in a MJ Mini™ Personal Thermal Cycler apparatus (Bio-Rad Laboratories, CA, USA). Melting curves were obtained by increasing the temperature from 60 to 95°C with a temperature transition rate of 0.5°C/sec. The comparative threshold cycle number (C_T_) method was used to assess the relative quantification of gene expression. The fold change of the target gene was calculated as 2^−ΔΔ^ C_T_ [18].

### Determination of reactive oxygen species (ROS)

Fluorimetric determination of intracellular ROS generated by adherent cells was performed in a 24-well plate after loading with 2′,7′-dichlorodihydrofluorescein diacetate (DCHF-DA) (Molecular Probes, Invitrogen, Paisley, UK), as previously described [19]. The intracellular ROS content was also measured by flow cytometry (Cyan). Harvested cells were incubated with 5 µM DCHF-DA for 20 min in the dark at 37°C, washed and re-suspended in PBS. For each sample, 10.000 events were acquired and intracellular ROS formation was detected as a result of the oxidation of DCHF at a wavelength of 520 nm. Data analysis was carried out with the Summit software.

Superoxide anion release was estimated by means of the superoxide dismutase-inhibitable cytocrome c reduction, as previously described [20]. Briefly, 15×10^3^ cells/well were plated in 24-well plates and incubated in Krebs-Ringer phosphate buffer with glucose (145 mM NaCl, 4.8 mM KCl, 0.5 mM CaCl_2_, 1.2 mM MgSO_4_, 5.7 mM NaPO_4_, 5.5 mM glucose [pH 7.4]) (KRPG) containing 80 µM ferricytochrome c (type III; Sigma), with or without SOD (final concentration 300 units/ml), in a total volume of 1 ml. After incubation for 1 h at 37°C, the reaction was stopped by placing the culture supernatants in melting ice, and the absorbance was read at 550 nm in a Sclavo Reader SR400 spectrophotometer (Siena, Italy). An extinction coefficient of 21×10^3^ M^−1^cm^−1^ was used for oxidized versus reduced cytochrome c.

### Western blot

Harvested cells were lysed in RIPA buffer (50 mM Tris-HCl pH 7.5, 150 mM NaCl, 1% NP40, 0.5% deoxycholate,0.1% sodium-dodecyl-sulfate, SDS) containing 2.5 mM Na-pyrophosphate, 1 mM β-glycerophosphate, 1 mM NaVO_4_, 1 mM NaF and a cocktail of protease inhibitors (Roche Diagnostic GmbH, Mannheim, Germany) for 30 min at 4°C. Cell lysates were centrifuged at 13.000 rpm for 10 min at 4°C and the concentration of cellular proteins was determined using the Bio-Rad assay (Bio-Rad). Cell proteins (20–30 µg) were mixed with a Laemmli buffer, separated on 12% SDS-PAGE, transferred onto a nitrocellulose membrane (Protan, Whatman GmbH, Dassel, Germany) and hybridized with the following antibodies according to the manufacturer's instructions: mouse anti-α-smooth muscle actin (SMA) mAb (1∶500; Sigma), mouse anti-TGF-β mAb (1∶500; BD Pharmingen, New Jersey, US), mouse anti-p-ERK-1/2 mAb (1∶1000; Santa Cruz Biotechnology, Santa Cruz, CA, USA), and mouse anti-phosphotyrosine (p-Tyr) mAb (1∶1000; Upstate, Lake Placid, NY, USA). Membranes were incubated with an horseradish peroxidase-conjugated anti-mouse secondary antibody (1∶2000; Amersham Pharmacia Biotech) and protein bands were revealed with the enhanced chemiluminescence (ECL) system detection kit (Roche). Immune-reactive bands were quantified by means of densitometry and normalized by mouse anti α-tubulin (1∶3000; Cell Signalling Technologies, Boston, MA, USA) mAb.

### Statistical analysis

Results are expressed as mean ± standard deviation (SD). All comparisons were made on a head-to head basis using the paired or unpaired *t* test, where appropriate. One way repeated measures ANOVA with *pot hoc* Bonferroni correction was used, where appropriate. A p value less than 0.05 was considered statistically significant. All analyses were performed with the GraphPad Prism 3 software package (GraphPad Software, San Diego, CA, USA).

## Results

### IPF Cells Display a Pro-Fibrotic Phenotype

**Figure 1:**
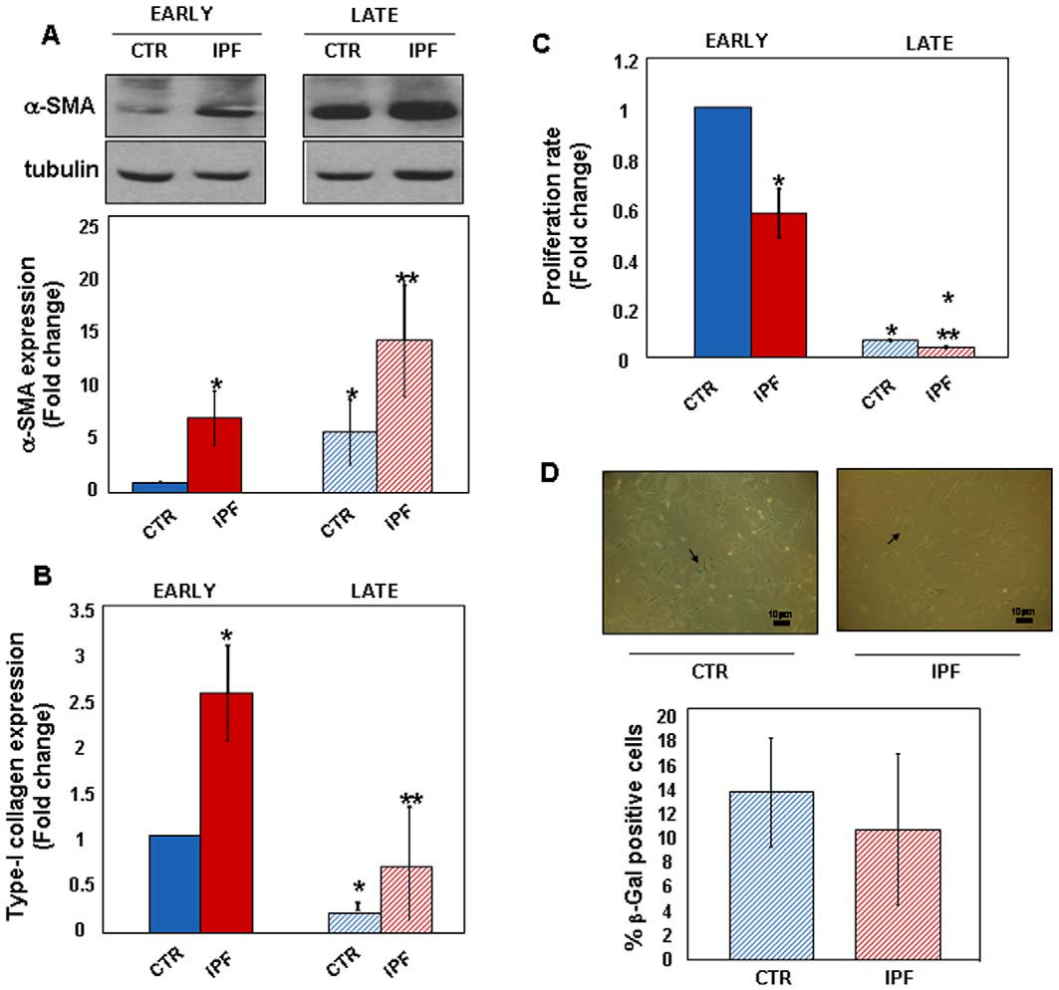
IPF Fibroblasts Display a Pro-Fibrotic Phenotype. The baseline phenotype of control (n = 4) and IPF cell lines (n = 7) grown in complete medium was analyzed at different culture passages. Cell lysates from sub-confluent cultures were immune-blotted with anti-α-SMA antibodies. Panel A shows a representative western blot of α-SMA (upper) and tubulin (lower) proteins in control and IPF fibroblasts at early and late passages. Relative statistics for early (full bars) and late (dashed bars) passage cells are also reported. *p<0.05 versus early passage control cells; ^**^p<0.05 versus early passage IPF cells. Panel B shows real time PCR expression levels of type-I collagen mRNA in control and IPF fibroblasts at early (full bars) and late (dashed bars) culture passages. *p<0.001 versus early passage control cells; **p<0.001 versus early passage IPF cells. Panel C shows the proliferation rate of early (full bars) and late (dashed bars) passage control and IPF fibroblasts. *p<0.001 versus early passage control cells; **p<0.001 versus early passage IPF cells. Data are expressed as mean fold change ± SD. Late passage control and IPF fibroblasts stained for senescence associated β-galactosidase activity are shown in panel D (magnification 10X). Arrows show β-galactosidase positive blue cells. The mean percentage of β-galactosidase positive cells ± SD in late passage cells (dashed bars) is reported.

**Figure 2:**
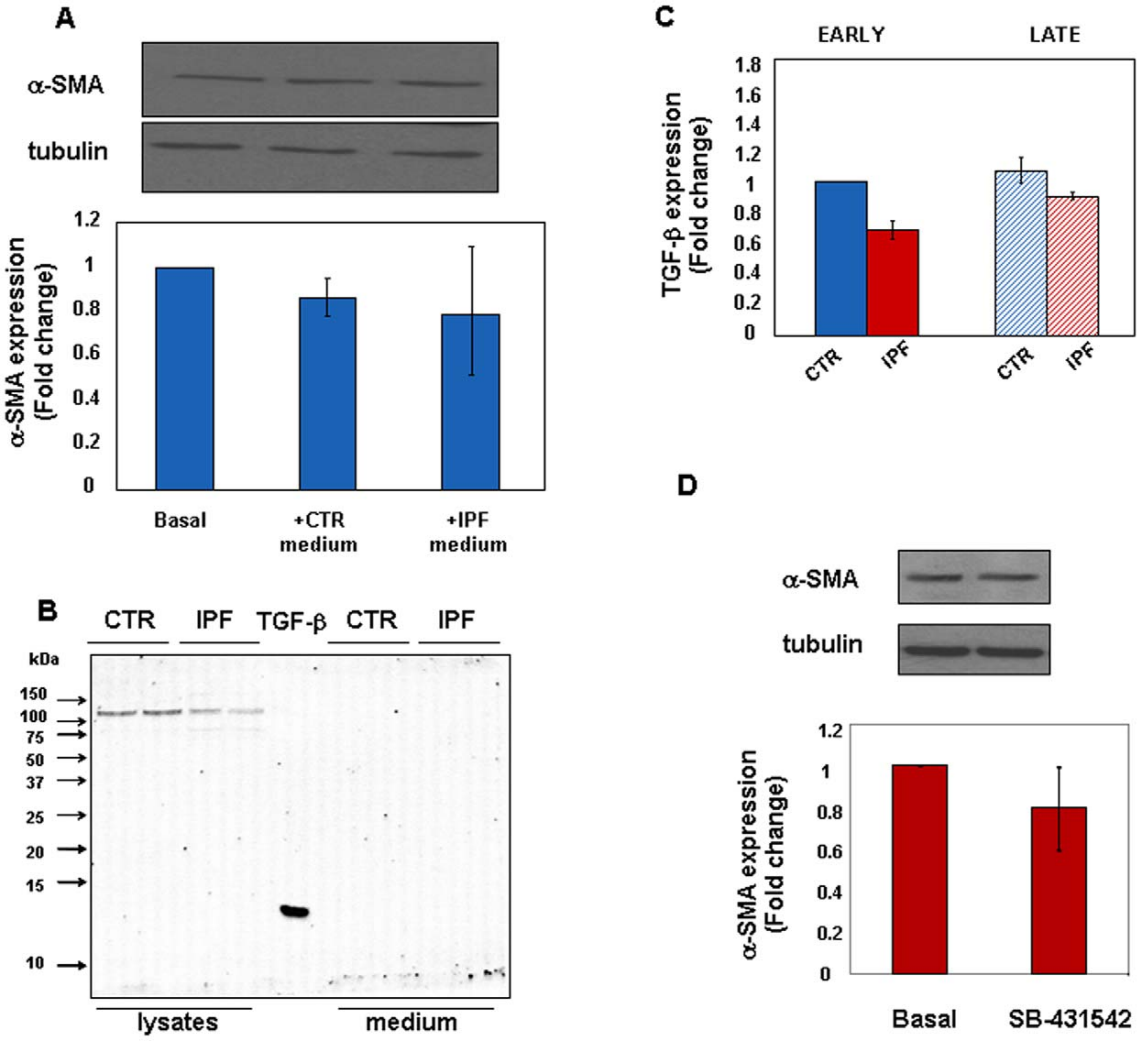
The IPF Phenotype is not Dependent upon the Autocrine Influence of TGF-beta. Early passage control fibroblasts (n = 3) were cultured in the presence of medium obtained from control or IPF cell cultures for 48 h. Panel A shows a representative western blot of α-SMA (upper) and tubulin (lower) proteins and relative statistics of three independent experiments. Representative immune-blots for TGF-β1 in cell extracts and culture media of early passage control and IPF fibroblasts are shown in panel B. Culture media were concentrated by means of centrifugal filter devices YM-10 (Centricon, Millipore). In the middle, 5 ng of rhTGF-β1 has been loaded as positive control. Panel C shows real time PCR expression levels of TGF-β mRNA in control (n = 4) and IPF fibroblasts (n = 7) at early (full bars) and late (dashed bars) culture passages. Early passage IPF fibroblasts (n = 3) were treated for 30 min with a specific TGF-β receptor ALK5 inhibitor (10 µM) and changes of α-SMA expression were assessed at the protein level. Representative immune-blots for α-SMA/tubulin expression and relative statistics are reported in panel D. All data are expressed as mean fold change ± SD.

To characterize the phenotype of IPF cells we measured the expression of the myofibroblast differentiation marker, α-SMA, and of type-I collagen. Expression of both molecules was increased in early passage IPF cells compared with control fibroblasts (Figure 1, A–B). Alpha-SMA expression increased in control and IPF fibroblasts with time in culture (Figure 1A), while that of type-I collagen was markedly reduced in late passage IPF cells and decreased further in control fibroblasts (Figure 1B). Proliferation of IPF cells, measured as DNA synthesis, was significantly less vigorous than that of control cells at early passages (Figure 1C). Proliferation significantly slowed down with time in culture in both cell types. However, expression of the senescence marker β-galactosidase was only barely detectable in both cell types with no substantial differences even at late passages (Figure 1D). Finally, to address whether the IPF phenotype was sustained by the ability of cells to produce growth factors, i.e. TGF-β, acting in an autocrine loop, α-SMA expression was analysed in early passage control fibroblasts conditioned for 48 h with the complete medium from control or IPF cell cultures. TGF-β is a pleiotropic cytokine that plays a crucial role in a wide array of processes, including wound healing and fibrogenesis. It is stored in the extra-cellular matrix as an inactive molecule which becomes activated after tissue injury. There is evidence that, among others, the integrin-mediated activation of TGF-β is pivotal in IPF pathogenesis [21]. As shown in Figure 2A, no significant differences in α-SMA expression occurred in the culture conditions used. These findings were not unexpected as integrin-mediated mechanisms were unlikely to have occurred in our experimental setting. Further, TGF-β protein levels were undetectable in both cell extracts and culture media from early passage control and IPF fibroblasts (Figure 2B). Also, no differences of TGF-β expression were appreciated at the mRNA level in control and IPF cells, both at early and at late passages (Figure 2C). Finally, sensitivity of early passage IPF fibroblasts to specific TGF-β receptor inhibition was assessed. As shown in Figure 2D, α-SMA expression was unchanged as compared to basal levels.

These data indicate that early passage IPF cells differ from control fibroblasts as: 1. are differentiated into myofibroblasts; 2. have a pro-fibrotic phenotype; and 3. proliferate less. Such a phenotype is not dependent upon the expression of growth factors, and especially of TGF-β, acting in an autocrine manner. Both cell types markedly slow down growth over time. This occurs along with myofibroblast differentiation with no evidence of ongoing senescence.

### Oxidative Stress Induces and Maintains the Phenotype of IPF Fibroblasts

**Figure 3:**
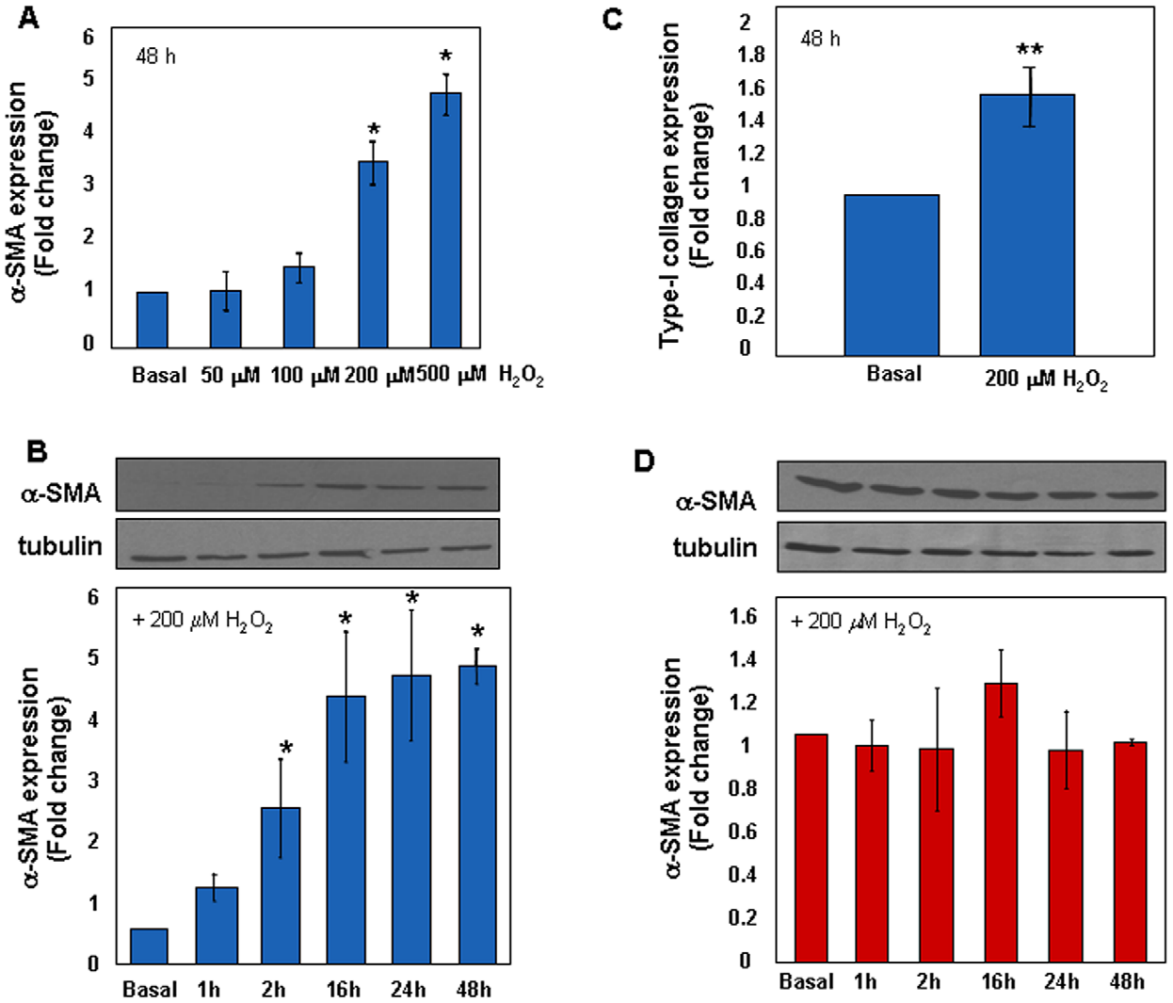
Chronic Oxidative Stress Induces a Pro-Fibrotic Phenotype. Changes of α-SMA expression were investigated by means of western blot in early control fibroblasts (n = 3) upon exogenous administration of hydrogen peroxide. Panel A shows changes of α-SMA expression upon cell treatment with increasing concentrations of H_2_O_2_ for 48 h. Conversely, panel B shows changes of α-SMA by treating cells with a fixed concentration of H_2_O_2_ (200 µM) at different time points. A representative immune-blot of α-SMA/tubulin is also shown. Panel C shows type-I collagen expression, assessed by real time PCR, in cells incubated for 48 h in the presence of H_2_O_2_ (200 µM). Expression of α-SMA in H_2_O_2_ (200 µM)-treated early passage IPF fibroblasts (n = 3) over time is shown in panel D. All data are expressed as mean fold change ± SD and are representative of three independent experiments. *p<0.001 versus basal; **p<0.05 versus basal.

**Figure 4:**
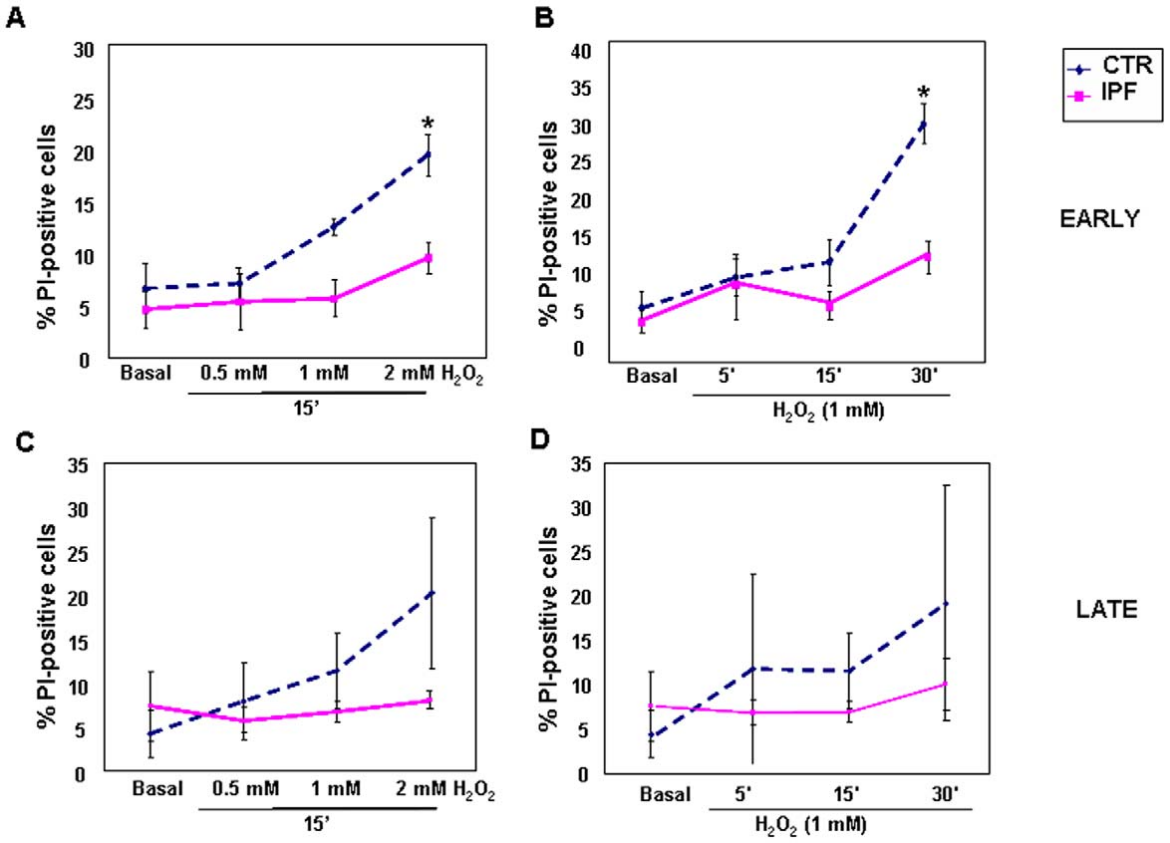
IPF Fibroblasts are Resistant to Acute Oxidative Stress-Induced Cell Death. Induction of cell death upon exogenous oxidative stress was assessed by means of flow cytometry in early and late passage control (n = 3) and IPF (n = 3) cells. Panels A and C show the distribution of the mean percentages of PI-positive cells treated for 15 min with increasing concentration of H_2_O_2_, in early and late passage cells, respectively. Panels B and D show the distribution of the mean percentages of PI-positive cells treated with a fixed H_2_O_2_ concentration (1 mM) at different time points in early and late passage cells, respectively. All data are expressed as mean percentage ± SD of fluorescent cells and are representative of three independent experiments. *p<0.05 versus control cells.

**Figure 5:**
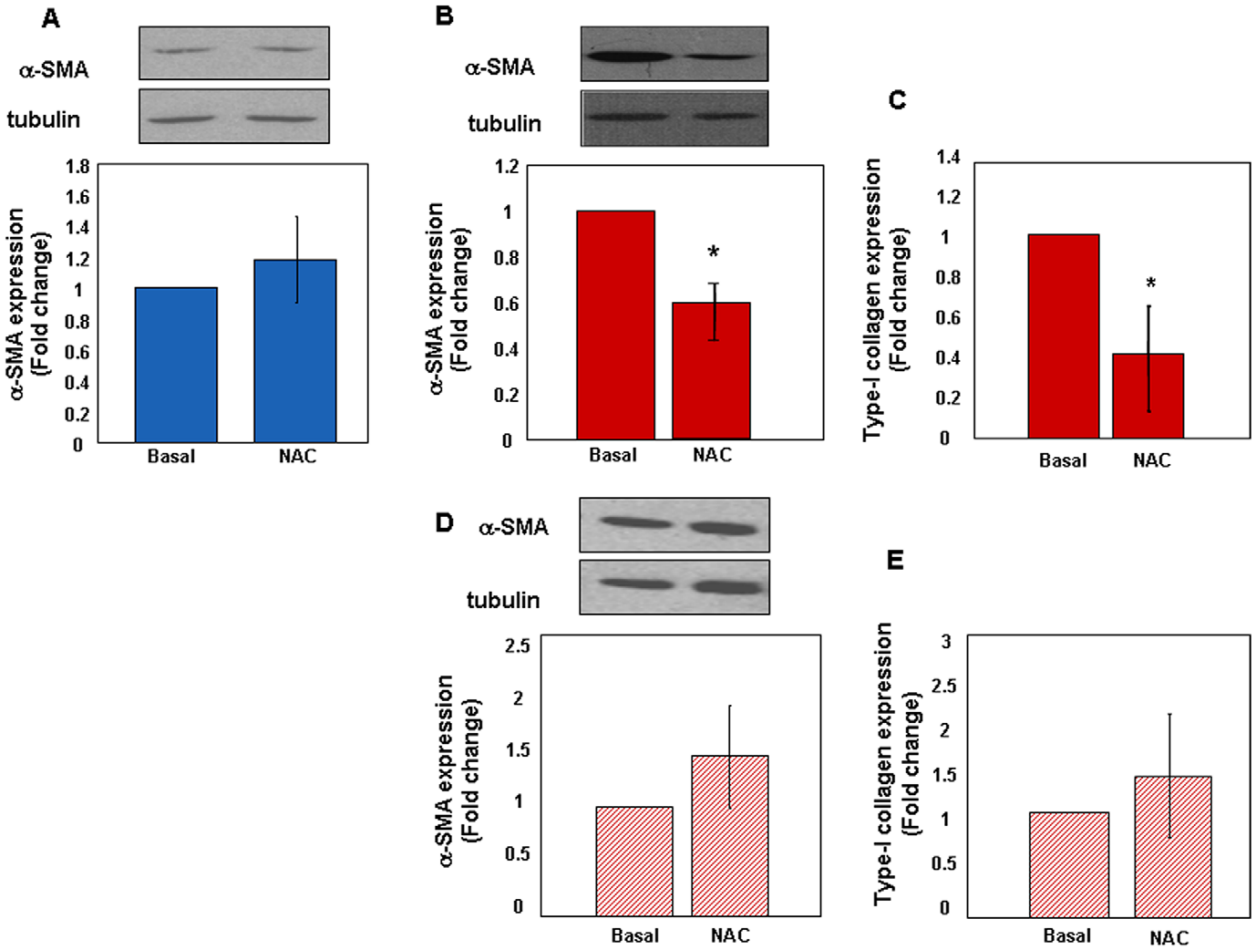
Negative Modulation of the Pro-Fibrotic Phenotype by ROS Scavenging. Control and IPF fibroblasts were treated for 48 h with the ROS scavenger NAC (5 mM). Panels A and B show representative immune-blots for α-SMA/tubulin and relative statistics in early passage control (n = 3) and IPF (n = 3) cells, respectively. Panel C illustrates changes of type-I collagen expression in early passage IPF fibroblasts. Changes of α-SMA and type-I collagen expression in NAC-treated late passage IPF fibroblasts (n = 3) are shown in panels D and E, respectively. Data are expressed as mean fold change ± SD and are representative of three independent experiments. *p<0.05 versus basal.

To investigate whether the phenotype of IPF cells was linked to oxidative stress we measured the expression of α-SMA and type-I collagen in control fibroblasts upon long-term challenge with H_2_O_2_. As shown in Figure 3A, α-SMA expression was robustly induced by H_2_O_2_ in a dose-dependent manner at 48 h. Time-course curves performed with a fixed concentration of H_2_O_2_ (200 µM) showed that induction of α-SMA expression increased over time, reaching the peak between 24 and 48 h (Figure 3B). Exposure to oxidative stress also accounted for higher expression of type-I collagen (Figure 3C). Under these conditions cell death was negligible, ranging from 8 to 13%. Conversely, as shown in Figure 3D, no further increase of α-SMA expression was appreciated in H_2_O_2_-treated early passage IPF fibroblasts. Short term incubation (15 min) with increasing concentrations of H_2_O_2_ induced death of early passage control fibroblasts in a dose-dependent manner (Figure 4A). Similarly, control cells death also occurred in response to a fixed concentration of H_2_O_2_ in a time-dependent manner (Figure 4B). Conversely, early passage IPF cells were resistant to peroxide-induced cell death in both culture conditions (Figure 4, panels A–B). IPF cells also retained their resistance to oxidative-induced cell death at later passages, as shown in Figure 4 (C–D). Finally, to test whether the IPF phenotype was sensitive to anti-oxidants, control and IPF fibroblasts were treated for 48 h with the ROS scavenger NAC (5 mM). Figure 5 (A–B) shows that levels of α-SMA were not modified in control fibroblasts while significantly decreased upon treatment in IPF cells at early passages. This finding was associated with a concomitant reduction of type-I collagen expression in IPF fibroblasts (Figure 5C). Conversely, no changes in α-SMA and collagen expression occurred in NAC-exposed IPF cells at late passages (Figure 5, panels D–E).

These data indicate that: 1. ROS stimulate normal fibroblasts to differentiate into type-I collagen expressing myofibroblasts; 2. conversely, ROS scavenging inhibits α-SMA and type-I collagen expression in IPF cells; and 3. myofibroblast differentiation of IPF cells confers resistance to oxidative stress-induced cell death.

### IPF Fibroblasts Are Characterized by High ROS Levels

**Figure 6:**
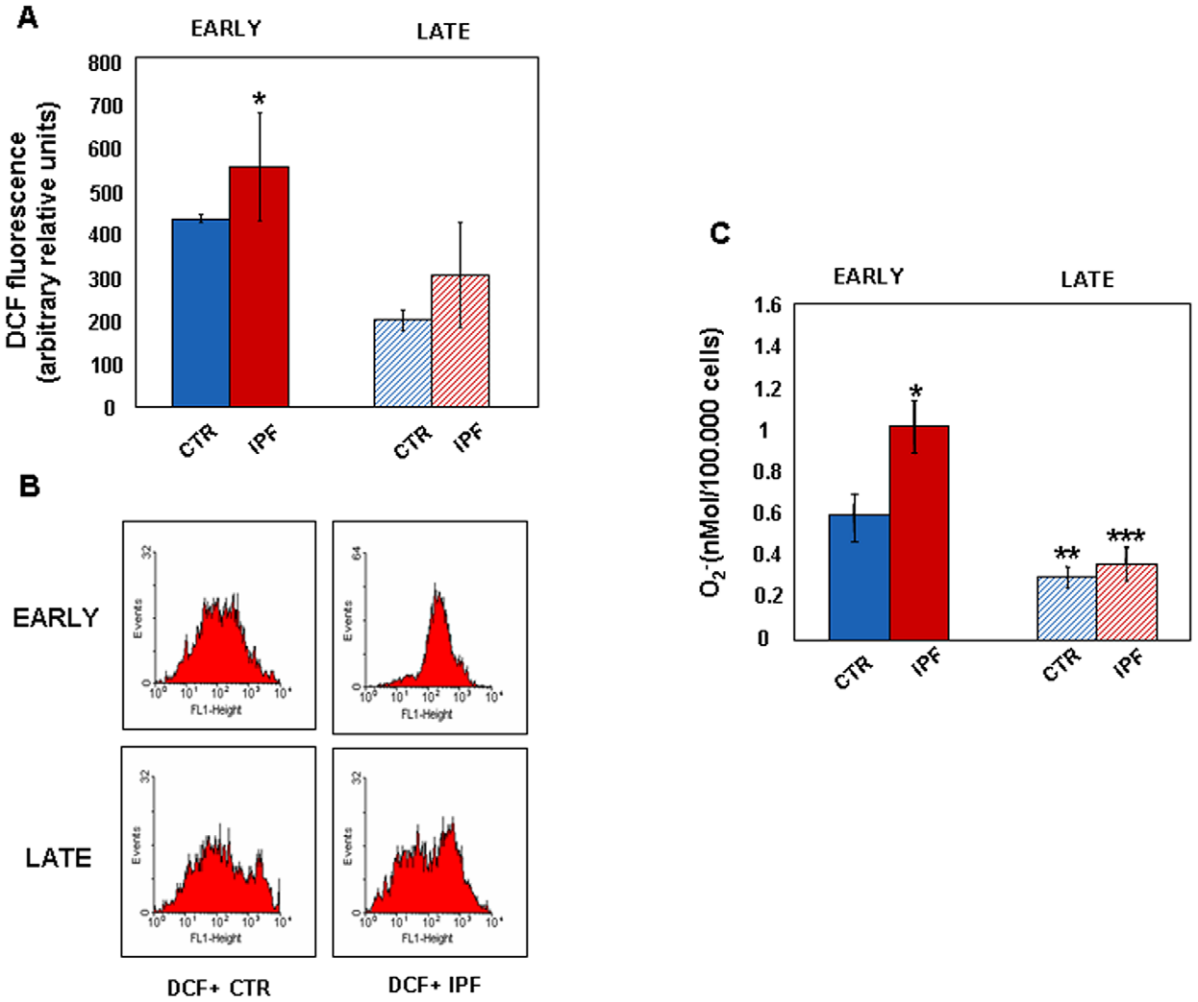
IPF Cells Produce High Levels of ROS. The baseline ability of control (n = 4) and IPF (n = 7) cells to generate ROS was measured at different culture passages with different methods. The intracellular content of ROS was first measured fluorimetrically in DCHF-DA loaded cells at early passages. Panel A shows levels of DCF fluorescence in early (full bars) and late (dashed bars) passage control and IPF cells. *p<0.05 versus control cells. Representative flow cytometry dot plots of the distribution of DCF fluorescence in control and IPF fibroblasts at different culture passages are shown in panel B. Panel C shows O_2_^-^ production, estimated by means of superoxide dismutase-inhibitable cytocrome c reduction, respectively in early (full bars) and late (dashed bars) passage control and IPF fibroblasts. All data are reported as mean value ± SD and are representative of three independent experiments. *p<0.01 versus early passage control cells; **p<0.05 versus early passage control cells; ***p<0.001 versus early passage IPF cells.

To dissect the nature of the signal(s) that characterizes the IPF phenotype, we measured the spontaneous ability of IPF and control fibroblasts to generate ROS. Mean basal ROS levels were significantly higher in early passage IPF fibroblasts compared with control cells, while no significant differences were recorded at late culture passages (Figure 6A). Flow cytometry analysis showed that early passage IPF fibroblasts were distributed as a cluster of highly fluorescent ROS-expressing cells (Figure 6B). Conversely, the distribution of fluorescent control cells analysed at the same culture passages was diffuse and heterogeneous. With time in culture the distribution pattern of ROS-expressing IPF cells became diffuse in a wider range of fluorescence similarly to control fibroblasts. To confirm the higher ability of IPF fibroblasts to generate ROS we measured the levels of superoxide anion released. Early passage IPF fibroblasts expressed higher superoxide anion levels compared with control cells (Figure 6C). No differences in superoxide anion release were found at late passages.

These data indicate that early passage IPF fibroblasts: 1. express high levels of ROS and superoxide anion in comparison with control cells; and 2. are undistinguishable from control cells with respect to ROS generation at late passages.

### ROS Generation in IPF Cells Occurs Through the Activation of a Membrane NADPH Oxidase-Like System

**Figure 7:**
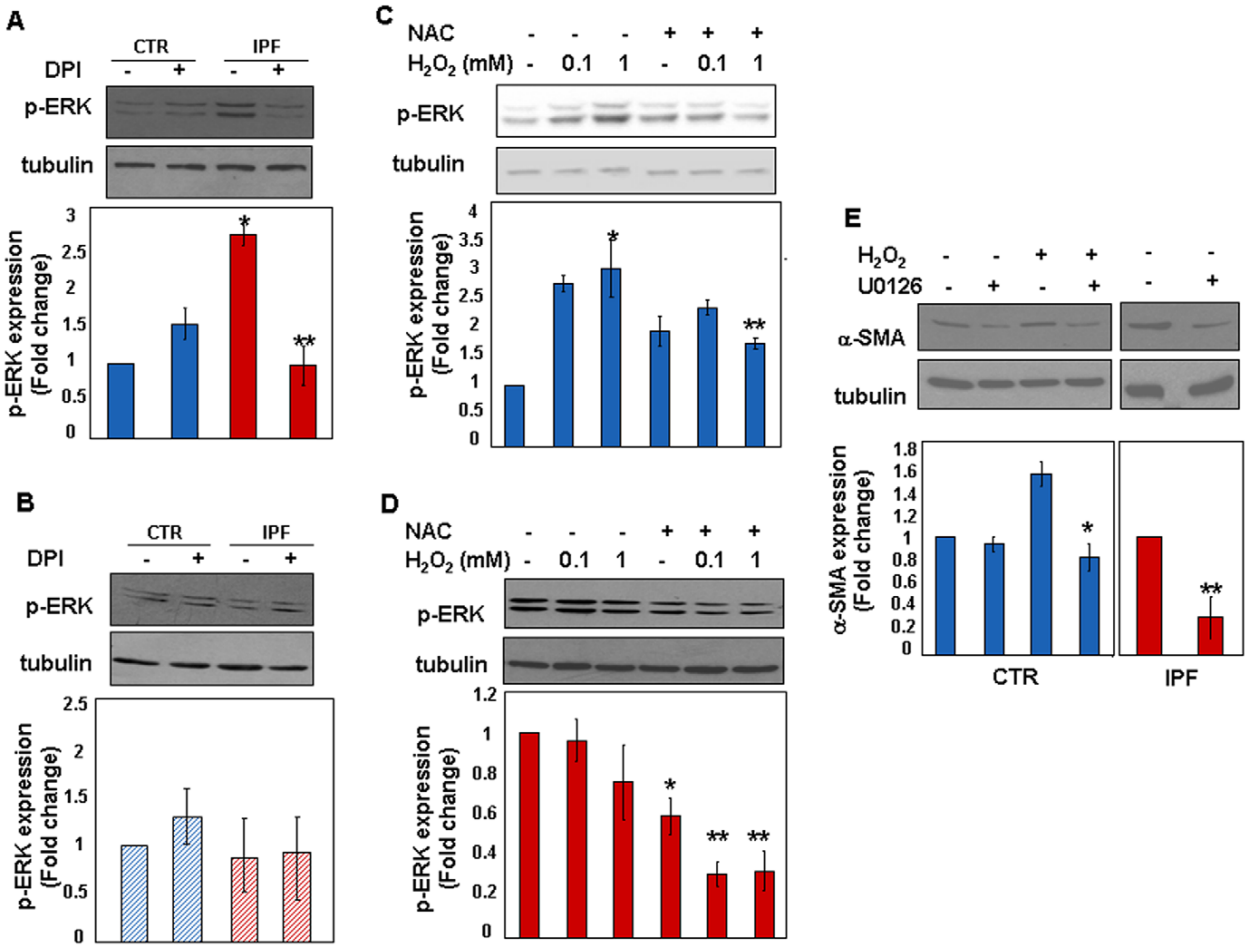
ROS Generation in IPF Fibroblasts Occurs Through the Activation of a Membrane NADPH Oxidase-Like System. Basal levels of ERK phosphorylation were assessed in control (n = 4) and IPF (n = 7) fibroblasts at different culture passages. Further, to determine whether a functional NADPH oxidase complex was involved in the generation of ROS, changes of p-ERK expression were analysed in response to treatment with DPI (20 µM), a flovoprotein inhibitor. Modulation of p-ERK levels was analysed upon 30 min of cell treatment. Representative immune-blots of p-ERK/tubulin and relative statistics are respectively shown in panels A and B. *p<0.001 versus basal control cells; **p<0.001 versus basal IPF cells. To investigate whether ERK signalling was inducible upon oxidative stress, early passage control (n = 3) and IPF fibroblasts (n = 3) were treated with increasing concentrations of H_2_O_2_ for 30 min. Pre-treatment of cells with the ROS scavenger NAC (10 mM for 1 h) was also analysed. Representative immune-blots and relative statistics of three independent experiments are reported in panels C (*p<0.01 versus basal; **p<0.01 versus 1 mM H_2_O_2_ treated cells) and D (*p<0.05 versus basal; **p<0.01 versus basal). To address whether ERK activation is necessary for α-SMA expression, modulation of peroxide-induced α-SMA (H_2_O_2_ 200 µM for 2 h) was analysed in early passage control cells (n = 3) pre-treated with UO126 (10 mM for 30 min). Similarly, α-SMA expression was evaluated in early passage IPF fibroblasts (n = 3) in response to UO126. Panel E shows representative immune-blots of α-SMA/tubulin and relative statistics. All data are expressed as mean fold change ± SD. *p<0.05 versus H_2_O_2_ treated cells; **p<0.05 versus basal.

To strengthen these observations we sought to identify the source of ROS. It is known that ROS activate MEK and ERK1/2 through the inhibition of tyrosine phosphatases [22], [23]: thus, ERK phosphorylation is inducible by ROS and ROS-induced p-ERK levels are sensitive to treatment with anti-oxidants or oxidase inhibitors. Basal levels of p-ERK were analysed in control and IPF fibroblasts at different culture passages. Then, since NADPH oxidase is the main source of O_2_^-^ in most cell types [24], changes of p-ERK expression were also analysed in response to cell treatment with the membrane NADPH oxidase inhibitor, DPI. As shown in Figure 7A, basal p-ERK expression was significantly higher in early passage IPF cells in comparison with the control ones. In addition, p-ERK levels significantly decreased in IPF fibroblasts in response to DPI. Conversely, control cells were insensitive to oxidase inhibition. No differences of p-ERK expression were detectable in control and IPF cells at late passages (Figure 7B). Similarly to control fibroblasts, IPF cells also became insensitive to DPI treatment. To further strengthen these observations, modulation of p-ERK levels upon H_2_O_2_ exposure was assessed in early passage control fibroblasts. As shown in Figure 7C, ERK phosphorylation was inducible in a dose dependent manner by H_2_O_2_ and this effect was inhibited by pre-treating cells with the ROS scavenger, NAC. Conversely, no further increase of p-ERK levels was detectable in response to H_2_O_2_ in early passage IPF cells that however were sensitive to NAC (Figure 7D). Finally, to address whether α-SMA expression was dependent upon ERK activation, modulation of peroxide-induced α-SMA expression was analysed in control fibroblasts pre-treated with the MEK kinase inhibitor UO126 (10 µM). As shown in Figure 7E, α-SMA was induced in response to H_2_O_2_ while was significantly decreased in UO126 pre-treated cells. Similarly, α-SMA expression did also decrease in IPF cells upon UO126 treatment.

These data indicate that IPF fibroblasts: 1. display an active NADPH-oxidase like system accounting for ROS formation; 2. contain active ERK1/2 which is an exquisite sensor of the activation status of such a system, and 3. α-SMA expression is maintained by ROS generation through ERK activation.

### Tyrosine Kinase(s) Signalling is Constitutively Activated in IPF Fibroblasts

**Figure 8:**
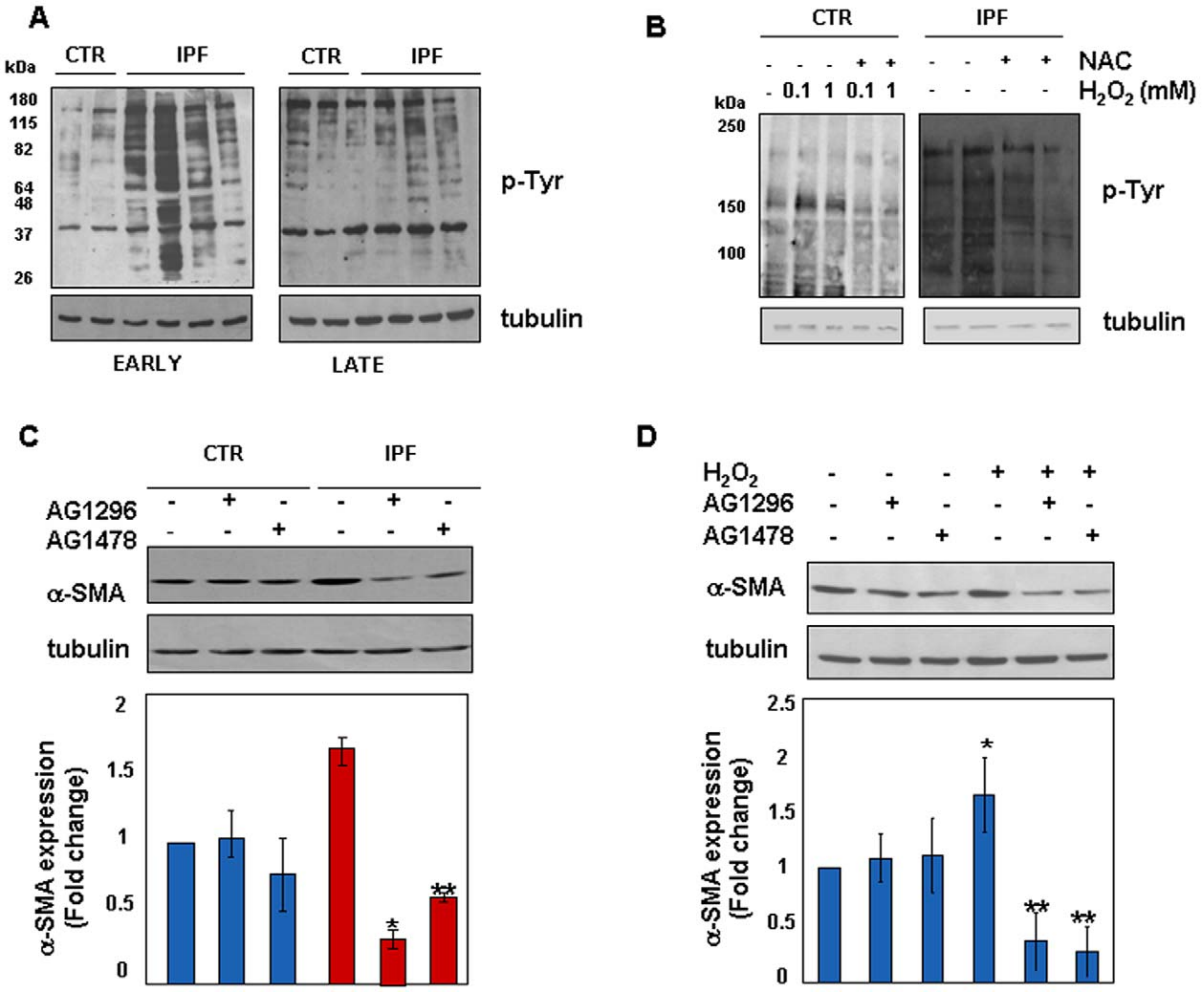
Constitutive Activation of Receptor Tyrosine Kinases in IPF Cells is Necessary for Differentiation into Myofibroblasts. Baseline levels of p-tyrosine proteins were assessed by western blotting in early and late passage control (n = 4) and IPF (n = 7) cells. Representative immune-blots are shown in panel A. Then, to address whether p-tyrosine signalling was inducible upon exogenous oxidative stress early passage control fibroblasts (n = 3) were treated for 15 min with increasing concentration of H_2_O_2_. Pre-treatment of cells with the ROS scavenger NAC (10 mM for 1 h) was also analysed. Modulation of p-tyrosine levels are shown in a representative immune-blot in panel B (left). Conversely, representative changes of p-tyrosine levels in early passage IPF cells (n = 3) treated with NAC (10 mM for 1 h) are shown on the right. To investigate whether myofibroblast differentiation is dependent upon tyrosine kinase signalling, changes of α-SMA expression were assessed by means of western blotting in early passage control (n = 3) and IPF (n = 5) fibroblasts treated for 30 min with the RTKs inhibitors AG1296 (anti-PDGF-R, 2 µM) and AG1478 (anti-EGF-R, 2 µM). Representative immune-blots of α-SMA/tubulin and relative statistics are shown in panel C. *p<0.001 versus untreated IPF; **p<0.01 versus untreated IPF. Finally, to investigate whether RTKs inhibition abrogates peroxide-induced α-SMA expression early passage control cells (n = 3) were pre-treated for 30 min with the RTKs inhibitors AG1296 (2 µM) and AG1478 (2 µM) and then exposed for 2 h to H_2_O_2_ (200 µM). Panel D shows representative immune-blots of α-SMA/tubulin and relative statistics. Results are expressed as mean fold change ± SD and are representative of three independent experiments. *p<0.05 versus basal; **p<0.001 versus H_2_O_2_ treated cells.

NADPH oxidase may be activated by a wide array of receptors for growth factors, including receptor tyrosine kinase(s) (RTKs) [25]–[27]. In addition, NADPH-generated ROS can themselves sustain signalling of RTKs through the inhibition of tyrosine phosphatases [22]. To test this issue, we asked whether ROS rise in IPF cells was associated with activation of tyrosine kinase(s) signalling. Figure 8A shows that IPF cells displayed higher basal levels of tyrosine phosphorylated proteins than control cells. Levels of tyrosine phosphorylation were decreased in IPF cells at late passages (Figure 8A). Tyrosine phosphorylation was inducible by H_2_O_2_ in control fibroblasts and this effect was inhibited by pre-treatment of cells with the ROS scavenger NAC (Figure 8B). In a similar manner, p-tyrosine levels also decreased in NAC treated IPF fibroblasts (Figure 8B). These findings are relevant as corroborate previous data indicating that switch-off of RTKs may have a beneficial effect in IPF through the inhibition of myofibroblast differentiation [28]–[30]. To address whether this was the case in our setting, we measured α-SMA expression in IPF cells upon inhibition of RTKs. Although we did not analyse the specific receptor involved, inhibition of RTKs was associated with a marked reduction of α-SMA levels in IPF cells. Conversely, α-SMA expression remained unchanged in control fibroblasts (Figure 8C). However, peroxide-induced α-SMA expression was sensitive to RTKs inhibition in control fibroblasts as shown in Figure 8D.

In conclusion, these observations suggest that tyrosine kinase(s) signalling: 1. is constitutively activated in IPF cells; 2. is inducible upon oxidative stress while, conversely, is inhibited by anti-oxidants; and 3. is required for myofibroblast differentiation.

## Discussion

Fibroblasts and myofibroblasts are key effectors in wound healing and are thought to be crucial players in IPF pathogenesis through an unabated continuation of the repair process after the resolution of inflammation [8]. ROS mediate multiple cellular functions, including proliferation, apoptosis, migration and differentiation [31], [32], all crucial events to tissue homeostasis and remodelling. Although ROS have been implicated in IPF pathogenesis, there is no conclusive evidence linking ROS metabolism to the phenotype of fibroblasts derived from IPF patients. To dissect the mechanism(s) underlying the disease, we first characterized the baseline phenotype of primary lung fibroblasts of IPF patients. Then, we focused on the redox state of IPF fibroblasts and investigated whether IPF features were influenced by oxidative stress. We found that, unlike control fibroblasts that predominantly showed a proliferative pattern, IFP cells were differentiated into myofibroblasts and displayed a pro-fibrotic phenotype. Previous studies have reported contrasting proliferative characteristics of human “fibrotic” fibroblasts [33], [34]. In our setting, IPF cells proliferated significantly less than control fibroblasts since the earliest culture passages corresponding to the first 20–25 PD. However, along with time in culture differences were no more appreciated as proliferation of IPF and control cells drastically slowed down. The absence of visible signs of senescence (β-galactosidase) which instead was associated with an increased α-SMA expression in both cell types over time in culture suggests that the progressive growth slow down was dependent on accelerated differentiation rather than on senescence [35]. IPF cells also displayed an increased ability to express higher levels of collagen expression than control fibroblasts at early passages. It is noteworthy that acquisition of the myofibroblast phenotype is necessary for collagen expression and that this effect is persistent and irreversible even after the removal of the inducing factor [36]. However, in our setting this ability was lost along with culture passages suggesting that the effect of the triggering factor was diluted out over time thus leading to the establishment of a quiescent phenotype.

In line with previous observations [37], IPF fibroblasts were committed to generate high ROS levels, and clustering of ROS-forming cells accounted for an increased amount of oxidants *per cell*. Interestingly, also the ability to produce ROS was lost with time in culture, as late passage IPF fibroblasts became undistinguishable from control cells. Altogether, these findings clearly suggest that IPF fibroblasts have a definitive functional lifespan and identify a precise temporal window to appreciate the differences between IPF and control fibroblasts.

As previously reported in skin fibroblasts obtained from systemic sclerosis patients [20], ROS generation in IPF fibroblasts occurred through the activation of a NADPH-like oxidase system. Indeed, levels of active ERK, which is an exquisite sensor of intracellular ROS rise, were susceptible to oxidase inhibition [19]. It has been recently shown that NADPH oxidase generation of H_2_O_2_ is required for TGF-β mediated fibrogenesis in a murine model of lung fibrosis and similar data have also been confirmed in human fibroblasts *in vitro* [38], [39]. In agreement with these observations we found that exogenous hydrogen peroxide strongly induced control fibroblasts to differentiate into type-I collagen expressing myofibroblasts. In addition, exposure to oxidative stress also accounted for activation of tyrosine kinase(s) signalling suggesting the existence of a link between such a pathway and cell differentiation. It is likely that the NADPH-related ROS rise in IPF cells derives from the constitutive activation of a wide array of receptors for growth factors, also including RTKs [25]–[27]. Although we did not look at the activation status of a specific tyrosine kinase receptor, we found that tyrosine kinase(s) signalling was activated in IPF cells and that RTKs activity was necessary for the maintenance of the IPF phenotype. There is evidence that in the presence of high ROS levels [19], [40] several receptors may be stabilized allowing cells to become more sensitive to sub-threshold concentrations of growth factors and cytokines. This sets on a circuitry linking growth factor receptors, including RTKs, NADPH oxidase and ROS, which self-maintains and propagates locally the inflammatory focus [19]. Far from being multiple discrete lesions, recent evidence suggests FF as a complex non-malignant reactive process responsive to local environmental stimuli. The formation of a fibroblast reticulum is consistent with a “wave” of fibrosis starting from the pleura and progressing through the lung parenchyma [41]. In this context, we suggest that the auto-amplificatory loop (RTKs, NADPH, ROS-pERK1/2) may be operating in the fibroblast reticulum where the progression of a “wave of oxidation” promotes through the freely ROS diffusion both cell to cell communication and myofibroblast differentiation.

In conclusion, the present study highlights the existence of a baseline distinctive molecular IPF phenotype characterized by an imbalanced redox state which self maintains in culture apart from the involvement of any triggering growth factor either exogenously added or acting in an autocrine manner. Although recent evidences suggest a role of autocrine TGF-β generation in maintaining the myofibroblastic phenotype in areas of fibrosis [42], we did not confirm these data in our setting. This phenotype confers resistance to oxidative stress-induced cell death, can be induced by ROS in control fibroblasts and is inhibited by ROS scavenging. Most importantly, the IPF trait is transient, since it is progressively lost along with time in culture. We suggest this measurable phenotype of IPF as an assay amenable for investigating the molecular signal(s) that initiate the events we described and for testing and validation of target specific therapies. It remains to be seen the site and the nature of the primary IPF trigger.
